# Reconstruction of gene regulatory network related to photosynthesis in *Arabidopsis thaliana*

**DOI:** 10.3389/fpls.2014.00273

**Published:** 2014-06-13

**Authors:** Xianbin Yu, Guangyong Zheng, Lanlan Shan, Guofeng Meng, Martin Vingron, Qi Liu, Xin-Guang Zhu

**Affiliations:** ^1^Group of Plant System Biology, CAS-MPG Partner Institute for Computational Biology, Shanghai Institutes for Biological Sciences, Chinese Academy of SciencesShanghai, China; ^2^Department of Computational Regulatory Genomics, CAS-MPG Partner Institutes for Computational Biology, Shanghai Institutes for Biological Sciences, Chinese Academy of SciencesShanghai, China; ^3^Department of Bioinformatics and Biostatistics, School of Life Sciences and Biotechnology, Shanghai Jiao Tong UniversityShanghai, China; ^4^Group of Plant System Biology, Institute of Plant Physiology and Ecology, Shanghai Institute for Biological Sciences, Chinese Academy of SciencesShanghai, China

**Keywords:** photosynthesis, transcription factors, regulatory network, small world, coordination

## Abstract

Photosynthesis is one of the most important biological processes on the earth. So far, though the molecular mechanisms underlying photosynthesis is well understood, however, the regulatory networks of photosynthesis are poorly studied. Given the current interest in improving photosynthetic efficiency for greater crop yield, elucidating the detailed regulatory networks controlling the construction and maintenance of photosynthetic machinery is not only scientifically significant but also holding great potential in agricultural application. In this study, we first identified transcription factors (TFs) related to photosynthesis through the TRAP approach using position weight matrix information. Then, for TFs related to photosynthesis, interactions between them and their targets were also determined by the ARACNE approach. Finally, a gene regulatory network was established by combining TF-targets information generated by these two approaches. Topological analysis of the regulatory network suggested that (a) the regulatory network of photosynthesis has a property of “small world”; (b) there is substantial coordination mediated by transcription factors between different components in photosynthesis.

## Introduction

In recent years, more and more research shows that improving photosynthetic efficiency is a major viable approach to further increase crop productivity for enhanced food and fuel production (Zhu et al., 2010). In this aspect, much research has been devoted to study the molecular mechanism of photosynthesis and identify potential options to further optimize the photosynthetic machinery (Zhu et al., 2010). A number of engineering targets have indeed been identified, such as increasing expression of SBPase (Lefebvre et al., 2005) and manipulation of recovery speed from photo-protected state (Zhu et al., 2004) etc. Great efforts are now undertaken to engineer these targets in different crops to improve photosynthetic efficiency.

In most of the current research on improving photosynthesis, individual components that can potentially increase photosynthesis were identified and used as targets for engineering. This approach has generated certain success, as in the case of SBPase where its over-expression increased photosynthesis and biomass production (Lefebvre et al., 2005; Zhu et al., 2007). However, this approach usually does not consider the inherent interaction between different components of photosynthetic machinery. Much evidence however suggested that there is substantial interaction among different components of photosynthesis. As a result, altering the expression level of one gene might generate changes in the expression level of many other photosynthetic genes simultaneously. For example, decrease of the expression level of Rubisco small subunit led to changes in phosphoribulokinase activity in Calvin-Benson cycle in tobacco (Hudson et al., 1992). Knock down of Rieske FeS led to decrease of the concentrations of the cytochrome b_6_f complex and Rubisco in tobacco (Price et al., 1998). In C4 plants, mutation of *Zmhcf136* caused loss of PSII complexes and grana thylakoid in mesophyll cells and simultaneous changes in expression patterns in the bundle sheath cells and mesophyll cells, including the differential levels of several C4 genes (e.g., PEPC, CA etc) (Covshoff et al., 2008). Over expression of C4 PEPC in rice also changed the expression level of Rubisco and FBPase (Agarie et al., 2002).

The close interaction among photosynthetic components is also reflected in the coordinated expressions of different components of photosynthetic machinery. In most C_3_ plants, surveys of photosynthetic physiological parameters showed that the maximal rate of Rubisco-limited photosynthesis (*V*_cmax_) and the maximal rate of RuBP-limited photosynthesis (*J*_max_) strongly correlated with each other (Wullschleger, 1993) suggesting that expression of the genes underlying these parameters should be highly coordinated. In maize, a typical C_4_ plant, the bundle sheath and mesophyll cells have distinct patterns of protein accumulation, e.g., compared to mesophyll thylakoids, the thylakoid in the bundle sheath cells have a 55% reduced PSII content, unchanged ATP Synthase content, and a 65% increased PSI and 33% increased cytb6f contents (Majeran et al., 2008). These stable coordinated expression of different components of photosynthesis in C_4_ plants also suggested that they were regulated by delicate regulatory networks.

The close coordination of expression of photosynthetic components has great physiological significance, in particular, it is critical for maintaining a high resource use efficiency of photosynthesis under different conditions. This has been demonstrated in a number of cases. For example, under elevated CO_2_ conditions, the expression level of soluble rbcs protein is gradually decreased in rice (Chen et al., 2005), which is consistent with the required adjustment for enhanced photosynthetic light and nitrogen use efficiency (Long et al., 2004). Therefore, understanding the molecular mechanisms underlying these coordinated changes in expression of different components in photosynthesis under different conditions will not only be of scientific significance but also have great agricultural application potentials.

Unfortunately, so far, only a limited number of transcription factors related to regulation of photosynthetic genes have been discovered, mainly through traditional forward genetic studies (Saibo et al., 2009). The research on construction of genetic regulatory network of photosynthesis using high throughput transcriptomic and genomic data are mostly lacking. This is in sharp contrast to the rapid progresses in construction of regulatory networks related to other plant processes, e.g., the circadian clock and flowering control (Keurentjes et al., 2007; Ma et al., 2007; Thai et al., 2007; Long et al., 2008; Lee et al., 2010).

In this study, we aim to construct genetic regulatory network using transcriptomic and genomic data. Specifically, we combined two existing bioinformatics algorithms, the TRAP (Roider et al., 2007) and the ARACNE (Basso et al., 2005; Margolin et al., 2006) approaches to construct gene regulatory network. We used *Arabidopsis thaliana* as the model species because its genomic sequences are available and there are large amount of transcriptomic data. The TRAP algorithm was developed to predict downstream target genes of TFs through calculating binding affinity between transcription factors and DNA fragments, which has been shown to have high accuracy in previous studies (Roider et al., 2007). ARACNE is a software developed to find assocition relationship between genes based on mutual information using expression data (Basso et al., 2005; Margolin et al., 2006). This study identified a number of new candidate transcription factors as regulators of photosynthesis, together with some TFs reported earlier. Though the resulting genetic regulatory network is still small-scale, the network already showed the “small world” property (Braha and Bar-Yam, 2004). We also found evidences suggesting that TFs played a crucial role in coordinating expression of different components in photosynthesis.

## Materials and methods

### Workflow of the whole project

**Figure 1:**
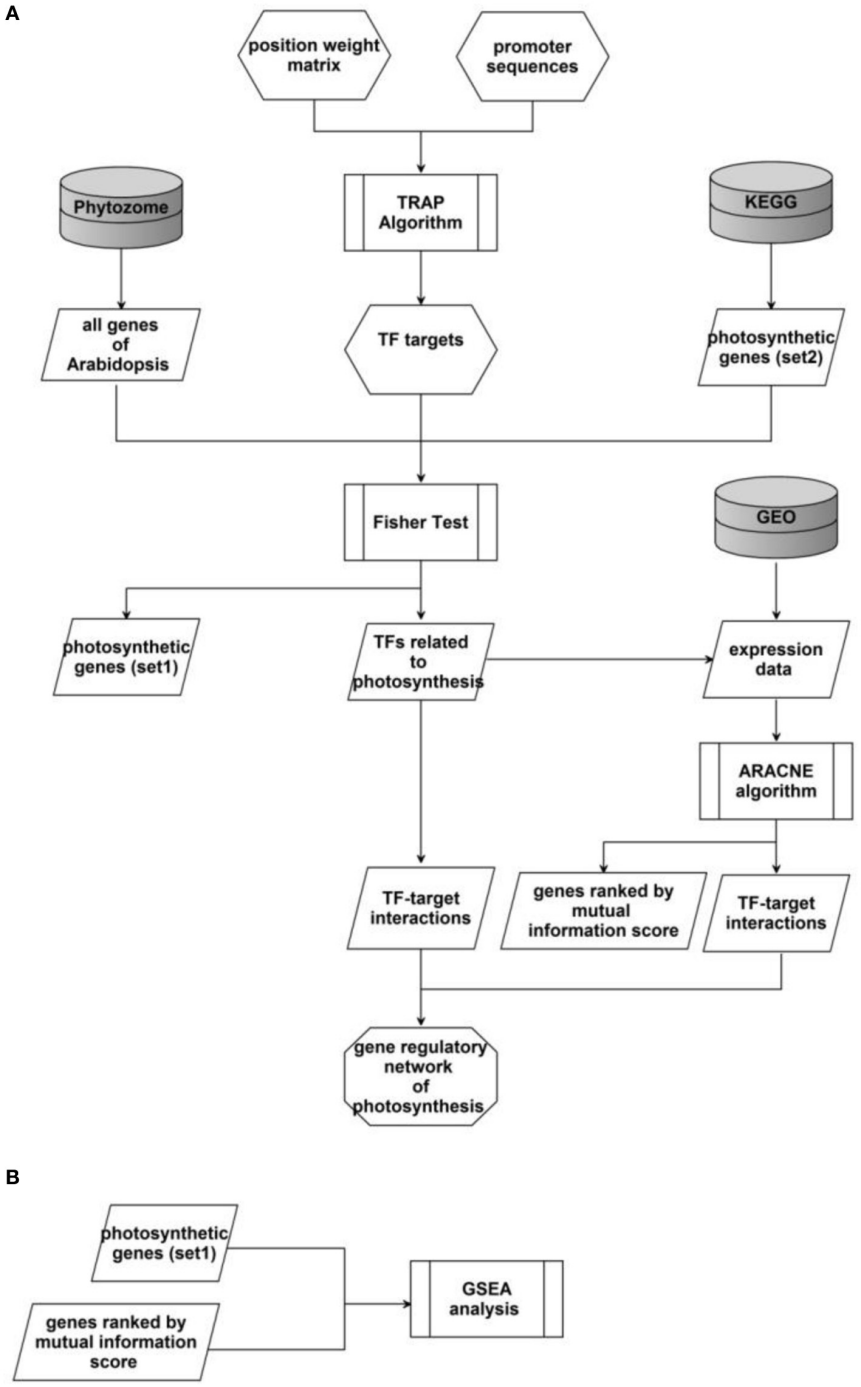
**Schema of the whole study**. **(A)** The workflow of the whole project. **(B)** The schema of GSEA analysis for a particular TF.

The workflow of our study was showed in Figure 1A. We first applied the TRAP algorithm to calculate the binding affinities between TF and DNA fragments in promoter regions of Arabidopsis genes. Then TFs involved in photosynthesis were identified through a modified Fisher's test. After that, we further identified the interaction between these TFs and their target gene using the ARACNE algorithm based on transcriptomics data. Finally, TF-target pairs identified by both algorithms were selected as edges of the gene regulatory network of photosynthesis in Arabidopsis. Here we describe in detail the major algorithms involved in this workflow.

#### Collecting and grouping photosynthetic genes

We first collected pathways (map00195 and map00710) associated with photosynthesis for Arabidopsis from the KEGG database (http://www.genome.jp/kegg/). The genes contained in these pathways were used as photosynthetic genes. These genes include all enzymes involved in the Calvin-Benson cycle, ATP synthesis, components involved in electron transfer, the light reactions and genes related to C_4_ photosynthesis. Though Arabidopsis did not operate C_4_ photosynthesis, all C_4_ photosynthesis related genes exist in Arabidopsis. Altogether, 124 photosynthesis-related genes or isoforms were used in our study. These genes were categorized into following groups according to their biological functions, i.e., the Calvin Cycle (CC), Photosystem II (PSII), Photosystem I (PSI), Light Harvest Complex (LHC), Photosynthesis Electronic Transport (PET), Cytb6/f, F-type ATPase (FTA), and C4 related genes (C4).

#### Predicting interactions between TFS and candidate genes using the TRAP algorithm

In this study, we defined the genomic region from upstream 1000 bp to downstream 500 bp from the transcription start site (TSS) of a gene as the promoter region. The promoter region sequences of all Arabidopsis genes were downloaded from the Phytozome database (http://www.phytozome.net). Then we collected all plant transcription factors and their corresponding position weight matrices (PWMs) from the TRANSFAC database (Matys et al., 2003). As a result, 124 TFs and their corresponding PWMs were obtained for further study. The promoter region sequences and PWMs were used as input of the Transcription Factor Affinity Prediction (TRAP) algorithm to predict interactions between TFs and candidate genes.

Given a PWM (with length *W*) of certain TF and a promoter sequence (with length *L*) of a potential targeting gene, the binding affinity score (N) is computed as the sum of contributions from all possible sites (l) in the promoter sequence through followed equation:

(1) $$(N)=\sum_{l=1}^{L-W}p_{l}=\sum_{l=1}^{L-W}\frac{R_{0}e^{-\beta E_{l}(\lambda )}}{1+R_{0}e^{-\beta E_{l}(\lambda )}}$$

Where, *p_l_* is the binding probability of site *l*, and *R*_0_ is defined by followed equation:

(2) $$R_{0}=K\;(S_{0})\;\cdot \;[TF]$$

Here, *K*(S_0_) is an equilibrium constant. In Equation (2), the energy *E* is set to be zero, and [TF] denotes the concentration of the transcription factor (Roider et al., 2007). Given a transcription factor, β E_l_(λ) describes its binding energies where the parameter λ is used to scale the mismatch energies in units of thermal energy (Roider et al., 2007).

Based on Equation (2), *R*_0_ is a constant for a TF at a given concentration. Therefore, we only need to consider the influence of parameter λ on binding affinity score. In practice, we used a series values of λ (i.e., 0.5, 0.6, 0.65, 0.7, 0.75, 0.8, 0.9, with 0.7 being the default value) to calculate the score. Given a certain value of λ, its impacts on the score was evaluated through counting the number of overlapped genes in top 1000 candidates compared with prediction result with a λ of 0.7.

#### Identifying transcription factors involved in photosynthesis

We first tested the impacts of modifying λ on prediction of TF and their targets. We found that varying λ did not influence the top 1000 genes much. Therefore, in this study, we used a λ value of 0.7. With this, we calculated binding affinities between these 124 TFs and their target genes with TRAP. Then, we used a modified Fisher's test to identify TFs whose target genes were significantly enriched in photosynthesis: (1) during calculation of the binding affinity scores, we set a series of values spanning from 0.5 to 5.0 with a step size of 0.1 as score cutoff. When a gene's binding affinity score was higher than the cutoff, it was regarded as a potential target of a certain TF. (2) A modified Fisher's test was conducted to calculate the *p*-value and judge whether targets were enriched in photosynthesis gene set among the background gene set. For each TF, a series of *p*-values were calculated based on different cutoffs values, and then the minimal *p*-value was selected. When the *p*-value was less than 0.001, the TF was regarded as a TF targeting photosynthesis genes.

#### Identifying TF-target pairs from microarray data

Microarray data of mature leaves in Arabidopsis (generated by the Affymetrix GPL198 platform) were downloaded from the GEO database (http://www.ncbi.nlm.nih.gov/geo/). Altogether 391 experiments with 5626 chips were obtained (Data Sheet 3). The ARACNE algorithm, which uses mutual information between TF and their target genes, requires differential expression for involved genes (Basso et al., 2005; Margolin et al., 2006). We therefore selected experiments in which more than half of the interested TFs have a relative high expression diversity, i.e., for which the coefficient of variation in the expression values is higher than 0.1. After this filtering step, we obtained 23 experiments with a total of 454 chips. These chips were normalized across experiments using “normalizeBetweenArrays” implemented in R package “limma” (Smyth, 2005). In this study, some TFs' PWMs were not derived from Arabidopsis. For these TFs, we identified their best orthologs in Arabidopsis through querying their sequences against the Arabidopsis protein database with the BLASTP program. After that, we used the ARACNE algorithm, which was developed to identify correlated gene pairs based on mutual information (Basso et al., 2005; Margolin et al., 2006), to identify TF-target pairs with a threshold *p*-value of 10^−7^. After that, for each TF, its target genes were put into a list, where genes were ranked in a descending order based on the value of mutual information.

#### Coherence analysis of two methods for photosynthetic genes

For a given TF, a ranked targeted gene list based on mutual information was constructed by the ARACNE algorithm. Then the software GSEA (Mootha et al., 2003; Subramanian et al., 2005) was utilized to detect whether the TF's target genes identified by the TRAP algorithm are enriched in this ranked gene list (Figure 1B).

#### Network properties of the regulatory network

The final genetic regulatory network was constructed by integrating the TF-target relationship predicted with both methods. We calculated a number of network properties for this network: (1) the degree of a node, which is the number of edges connected to node; (2) the diameter of a network, which is the longest value of all the shortest paths in the network; (3) clustering coefficient of a node, which is the ratio of existing edges linking a node's neighbors to each other to the maximum possible number of such edges among the neighbors of a node.

## Results and discussion

### A feasible strategy for identification of TFS related to photosynthesis

**Figure 2:**
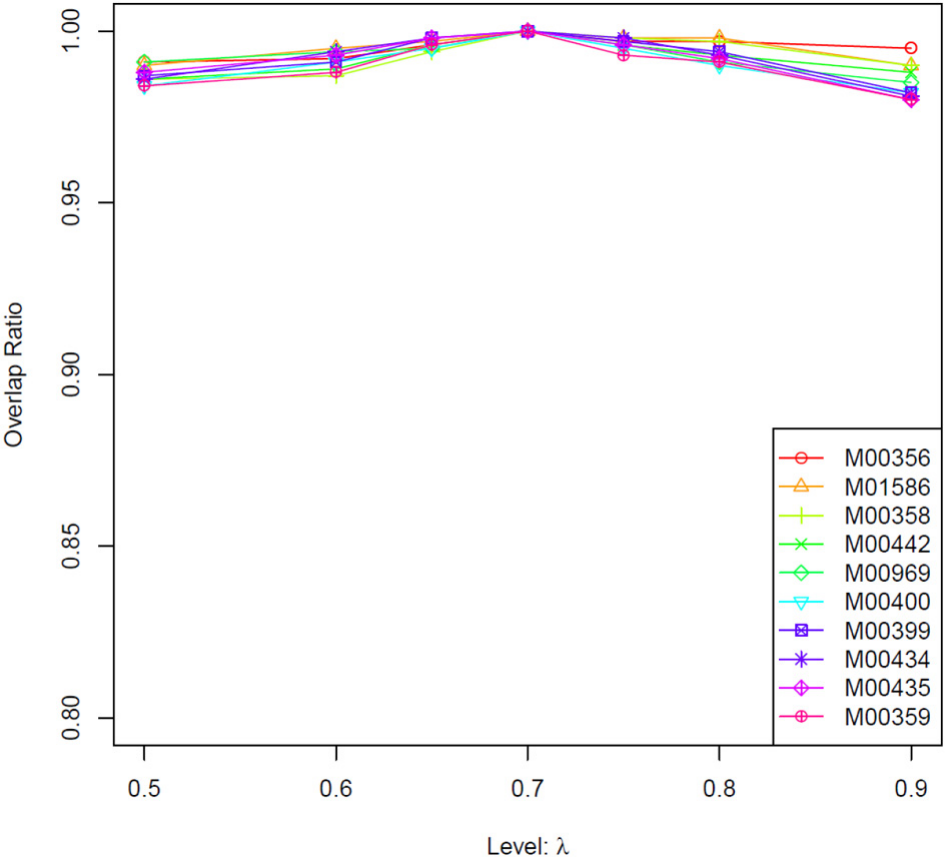
**Comparison results of different values of parameter λ for the TRAP algorithm**. We used a number of values of λ, i.e., 0.5, 0.6, 0.65, 0.75, 0.8, 0.9, to calculate the binding affinity between TFs and all the genes in Arabidopsis genome using TRAP. Then for each TF, we chose the top 1000 target genes with high binding affinity. We further calculated the overlap between this gene set and the top_1000_genes identified under λ = 0.7 (the default value). The horizontal axis denotes the used values of λ, while vertical axis denotes the ratio of overlap between these two gene sets. The result shows that TRAP is not sensitive to the parameter value of λ. Here the overlaps for only two TFs' results are showed (see the Supplemental Table 1 for all results). M00503, ATHB-5, from *Arabidopsis thaliana*; M00356, bZIP910, bZIP transcription factor from Antirrhinum majus.

In this study, we first tested whether prediction results of the TRAP algorithm is sensitive to parameter λ. As shown in data sheet 1 (see Supplemental Data), for most of the transcription factors, more than 90% of the top 1000 genes generated by different λ value were identical. Results of ten selected TFs are showed in Figure 2. These results suggest that predictions from the TRAP algorithm is not sensitive to the parameter λ. Hence, in this study, we use the default value (0.7) of λ to calculate the binding affinity between TFs and candidate genes in Arabidopsis. Subsequently, a modified Fisher's test is utilized to identify TFs related to photosynthesis (*p*-value < 0.001). In total, we identified 13 TFs that were related to photosynthesis (Table 1), among which 8 TFs have been reported earlier to have close relation to the regulation of photosynthesis (Table 1). Additionally, RITA-1, although it was not reported as a regulator of photosynthesis, its ortholog in Arabidopsis, bZIP9, can form a heterodimer with bZIP25 (Opaque-2) and bZIP63 (CPRF-2), which play an important role in regulation of light-induced genes (annotated in UniproKB) (http://www.uniprot.org). So it is possible that RITA-1 is also an important regulator of photosynthesis. Therefore, the strategy using in our study to recognize TFs involved in photosynthesis is feasible, since 9 out of 13 TFs (around 70%) are verified as important regulators of photosynthesis.

**Table 1:** **Summary of TFs related to photosynthesis**.

**TF**	**Species**	**PWM**	**Function**	**Homolog in Arabidopsis**
Opaque-2	*Zea mays*	NCCACGTVRN	Activation of cyPPDK1; additive increase in combination with PBF	BZIP25(AT3G54620)
CPRF-1	*Petroselinum crispum*	KMCACGTGKM	Regulate light-induced genes	GBF3(AT2G46270)
CPRF-3	*Petroselinum crispum*	NHSACGTSDN	Regulate light-induced genes	GBF1(AT4G36730)
RITA-1	*Oryza sativa*	YSACGTR	The rice bZIP transcriptional activator RITA-1 is highly expressed during seed development	BZIP9(AT5G24800)
EmBP-1	*Zea mays*	GCCACGTGAN	Can activate transcription from a truncated promoter containing a pentamer of the O2 site in yeast cells;	GBF1(AT4G36730)
CPRF-2	*Petroselinum crispum*	NHCACGTGDN	Regulate light-induced genes	BZIP63(AT5G28770)
TGA1b	*Nicotiana tabacum*	DHSACGTSDB	Binds specifically to the DNA sequence 5′-TGACG-3′	AT2G40950
HBP-1a	*Triticum aestivum*	GNCACGTGGC	Binds to the hexamer motif 5′-ACGTCA-3′ of histone gene promoters	BZIP16(AT2G35530)
TAF-1	*Nicotiana tabacum*	GCCACGTGGC	Binds to a G-box-related element, (5′-GCAACGTGGC-3′). Also binds to the HEX-motif of wheat histone H3 promoter	GBF3(AT2G46270)
PIF1	*Arabidopsis thaliana*	GNCACGTGRN	Regulates negatively chlorophyll biosynthesis and seed germination in the dark, and light-induced degradation of PIF1 relieves this negative regulation to promote photomorphogenesis	AT2G20180
GBF1	*Arabidopsis thaliana*	TKCCACGTGGCM	Binds to G-box motif (5′-CCACGTGG-3′) of rbcS-1A gene promoter. Regulate light-induced genes	AT4G36730
GAMYB	*Hordeum Vulgare*	NNSCRRYAACNVA	Transcriptional activator of gibberellin-dependent alpha-amylase expression in aleurone cells	MYB33(AT5G06100)
ARR10	*Arabidopsis thaliana*	AGATHYK	Functions as a response regulator involved in His-to-Asp phosphorelay signal transduction system	AT4G31920

*The annotation information of transcription factors are collected from the TRANSFAC and the UniProt database*.

### Coherence analysis of photosynthetic gene sets

After identifying TFs related to photosynthesis by the TRAP algorithm, homologies of these TFs in Arabidopsis were collected through the BLASTP program in NCBI (www.ncbi.nlm.nih.gov). As a result, 10 TFs in Arabidopsis and their corresponding targets (set1) were obtained. For each of these 10 TFs, we used the ARACNE software (Basso et al., 2005; Margolin et al., 2006) to calculate mutual information between the TF and all genes in Arabidopsis. Then genes associated with the TF were ranked based on the mutual information score. After that, for each TF, a coherence analysis is conducted between targets identified by the TRAP and the ranking generated by the ARACNE software (Table 2). As showed in the Table 2, genes of set1 are significantly enriched on top of the rankings for almost TFs except HBP-1a. These results suggested that the TFs regulating photosynthetic gene sets identified by TRAP and ARACNE were consistent with each other.

**Table 2:** **Results of coherence analysis for TF targets identified by the TRAP and ARACNE algorithm**.

**TFs**	**Homologs**	**TF targets (set1)**	
		**Num**	**NOM *p*-val**
PIF1	AT2G20180	26	0.23673469
**HBP-1a**	**AT2G35530**	**12**	**0.82678**
TGA1b	AT2G40950	23	<0.001
CPRF-1(TAF1)	AT2G46270	30	<0.001
Opaque-2	AT3G54620	28	0.002079002
ARR10	AT4G31920	5	0.08054523
CPRF-3,EmBP-1,GBF	AT4G36730	43	<0.001
GAMYB	AT5G06100	67	0.008008008
RITA-1	AT5G24800	28	0.15663901
CPRF-2	AT5G28770	53	0.069

### Identified TFS related to photosynthesis regulation

Table 1 lists the TFs identified by the TRAP algorithm. Among these TFs, many belong to the family of G-box binding protein [22]. G-box is a *cis*-acting element, 5… CACGTG… 3, identified in promoters of many plant genes (Williams et al., 1992). G-box binding proteins is a big gene family, while CPRF-1, CPRF-2, CPRF-3, EmBP1, TAF1, Opaque-2, GBF1 are members of the family (Siberil et al., 2001) (Table 1). CPRF-3 and EmBP1 are homologs of Arabidopsis GBF1 while CPRF-1 and TAF-1 are homologs of Arabidopsis GBF3 (Table 1). GBF1 exists in the nuclei of tomato and Arabidopsis and can interact with the G-box motif in promoters of many rbcs isoforms; while GBF3 shares the same binding motif with GBF1 (Giuliano et al., 1988). Earlier reports have shown that GBF1 and HY5 form DNA-binding heterodimer at *rbcs-1a* promoter; but different from HY5, GBF1 is a negative regulator of *rbcs-1a* [24]. GBF1-HY5 heterodimer is also a positive regulator of CAB1, which promotes accumulation of chlorophyll in cells and plays an important role in blue-light-induced photo-morphogenesis (Singh et al., 2012). Previous study also suggested that in Arabidopsis the Pro-rich activation domain of GBF1 can interact with GLK2 and GLK1, which regulates chloroplast development in diverse plant species (Tamai et al., 2002). These evidences suggested that GBF1 is an important TF regulating photosynthesis. Another identified G-box binding protein CPRF-2 can form heterodimer with CPRF1 or CPRF3 (Armstrong et al., 1992). Therefore, these 6 G-box TFs are potentially important regulators of photosynthesis. Opaque-2 (O2), another G-box binding factor, is also identified as an important regulator controlling expression of photosynthetic genes (Table 1). Opaque-2 has been reported to control the expression of a cytosolic form of pyruvate orthophosphate dikinase-1 (cyPPDK1) (Maddaloni et al., 1996), a key enzyme in the C4 photosynthesis. In the *o2* mutant maize, expression of multiple photosynthetic genes were are down-regulated, included PEPC and ME (Hartings et al., 2011). By the way, EmBP-1 can bind to the same site as Opaque-2 in the same promoters, but inhibit the regulated transcription of these promoters of Opaque-2. (Carlini et al., 1999). We also identified PIF1, which is a negative regulator of chlorophyll biosynthesis and seed germination in darkness, light-induced degradation of PIF1 can relieve this negative regulation and promote photomorphogenesis (Moon et al., 2008).

### Properties of the gene regulatory network of photosynthesis

**Figure 3:**
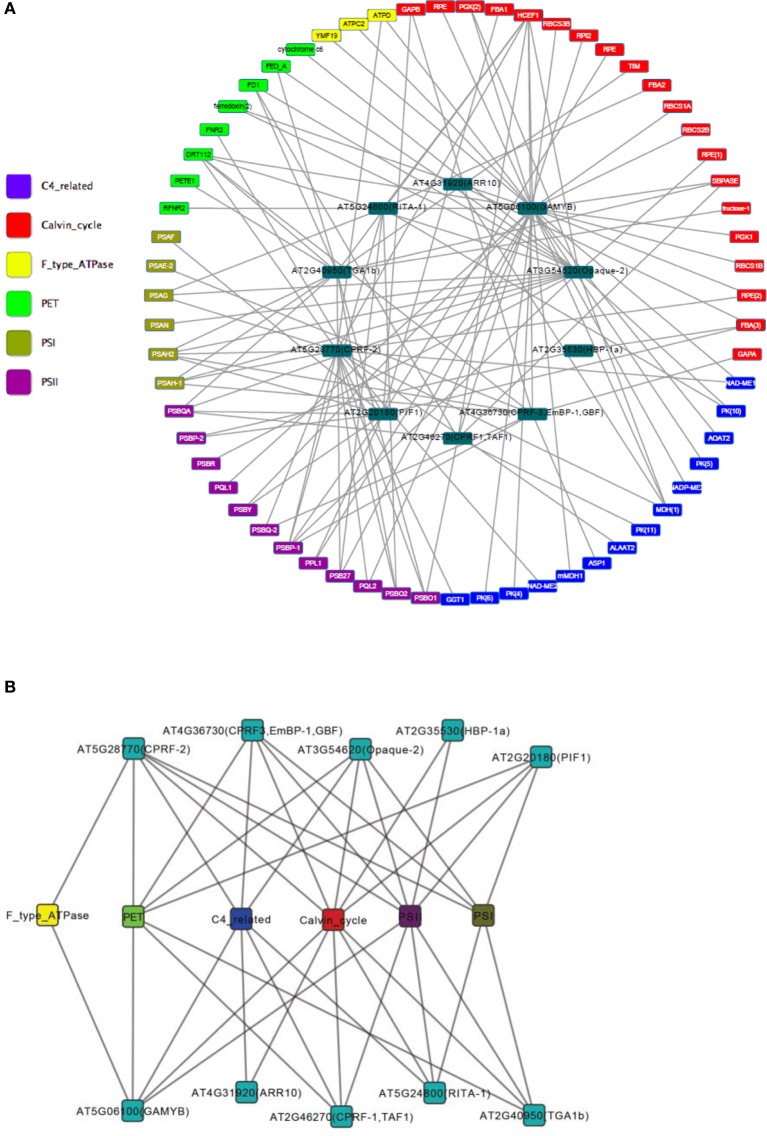
**Gene regulatory network of photosynthesis in Arabidopsis**. **(A)** Nodes in different colors represent genes involved in different sections of photosynthesis. We have divided the photosynthesis system into the following sections: ATPase, enzymes related to C4 photosynthesis, Calvin cycle, light harvesting complex, photosynthetic electron transfer, photosynthesis I, and photosystem II. **(B)** Transcription factor-components network. Photosynthesis genes are assigned to components. And a transcription factor and a component are linked if the transcription factor linked to one of the genes in the component in network **(A)**.

**Figure 4:**
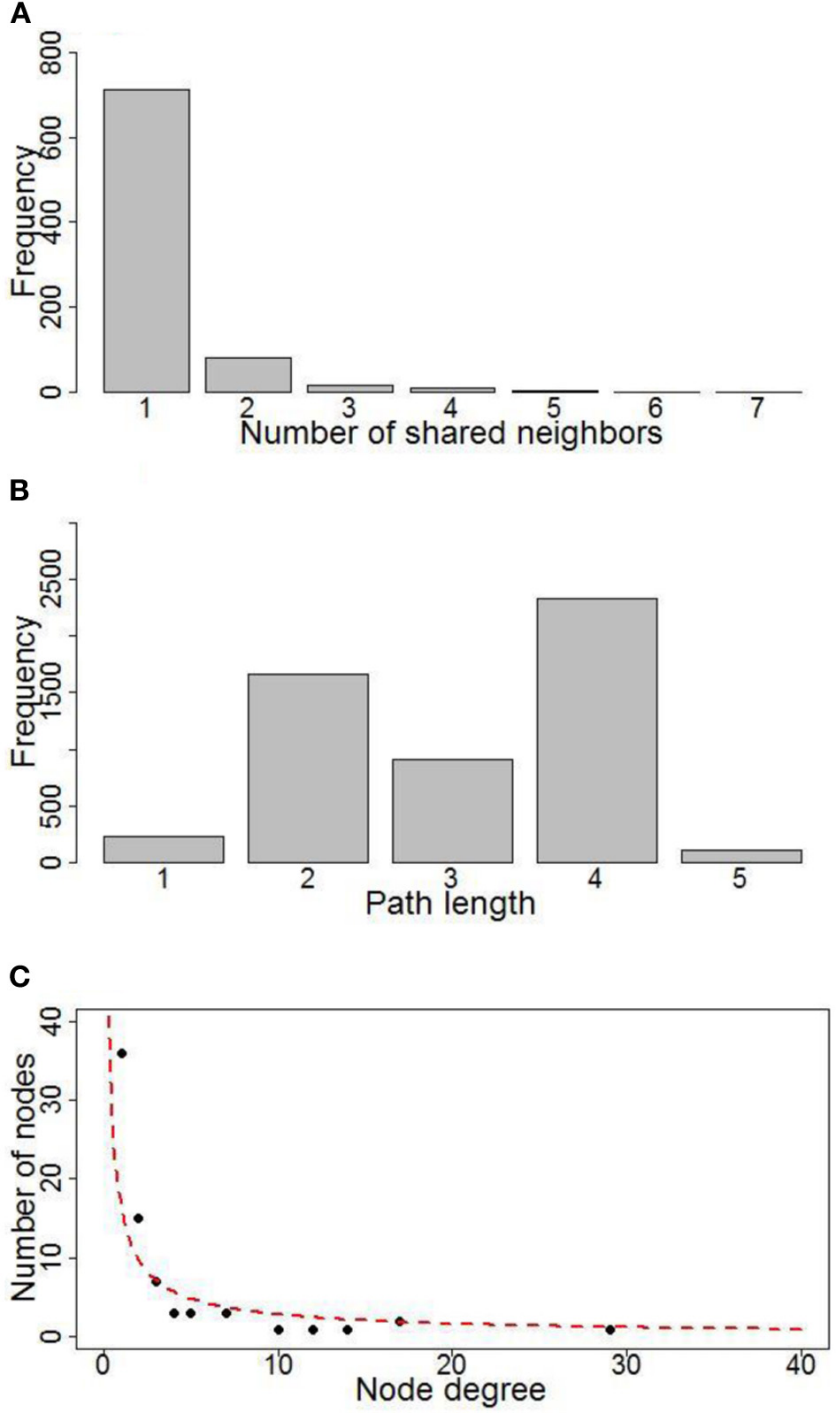
**Topology property of the photosynthetic network. (A)** Distribution of neighbors for nodes; **(B)** Distribution of shortest path length for the network; **(C)** Plot of number of nodes VS. nodes' degree.

A gene regulatory network of photosynthesis was established by combining TF-target pairs identified by both the TRAP and the ARACNE algorithm (Figure 3A). Then topology analysis was conducted by the “NetworkAnalyzer” module (Assenov et al., 2008) of the cytoscape software (http://www.cytoscape.org/). Topology analysis of the network showed that it has a diameter of 6; the average number of neighboring nodes and average length of shortest path are 4 and 3 respectively (Figures 4A,B). In addition, distribution of node degree in the network follows a power-law distribution (Figure 4C). These results indicate that the photosynthetic network is a scale free network, i.e., most of the components in the photosynthesis are regulated by relatively small number of regulators, i.e., TF in this case. These small number of regulators are regarded as hubs of the photosynthetic regulatory network and may play crucial role in coordination of expression of genes involved in photosynthesis. In the regulatory network that we obtained, the components of PSI, PET and PSII parts are co-regulated by the TFs of TGA1b, PIF1, Opaque-2, and CPRF-2 (Figure 3B). Furthermore, genes from Calvin cycle and F-Type ATP synthase are also co-regulated TF GAMYB and CPRF-2 (Figure 3B). Detailed reverse genetics studies should be conducted to study these coordination and their physiological significance.

## Conclusions

In this study, a genetic regulatory network of photosynthesis in Arabidopsis was constructed through combining the genomic sequence, TF binding information and gene expression data. We identified a number of novel transcription factors related to photosynthesis. The identified network follows a scale-free property. The potential hubs in the network coordinating various components of photosynthesis were also identified. Transgenic experiments are undergoing now to test the consequence of manipulating expression of these TFs on photosynthetic performances.

### Conflict of interest statement

The authors declare that the research was conducted in the absence of any commercial or financial relationships that could be construed as a potential conflict of interest.
