# Supplementary material for: Reconstruction of gene regulatory network related to photosynthesis in Arabidopsis thaliana
Source: Front Plant Sci. 2014 Jun 13;5:273. doi: 10.3389/fpls.2014.00273 (PMC4055858; doi:10.3389/fpls.2014.00273)
Supplement: Supplementary file 3 [file DataSheet3.PDF]

"gse.name"
"1" "GSE10016"
"2" "GSE10019"
"3" "GSE10039"
"4" "GSE10247"
"5" "GSE10248"
"6" "GSE10322"
"7" "GSE10323"
"10" "GSE10414"
"11" "GSE10464"
"12" "GSE10496"
"13" "GSE10497"
"14" "GSE10501"
"15" "GSE10502"
"16" "GSE10509"
"17" "GSE10522"
"18" "GSE10568"
"19" "GSE10576"
"20" "GSE10643"
"21" "GSE10646"
"22" "GSE10670"
"23" "GSE10719"
"24" "GSE10732"
"25" "GSE10749"
"26" "GSE10801"
"27" "GSE10812"
"28" "GSE10876"
"29" "GSE10928"
"30" "GSE11119"
"31" "GSE11216"
"32" "GSE11250"
"33" "GSE11262"
"34" "GSE11505"
"35" "GSE11532"
"36" "GSE11558"
"37" "GSE11594"
"38" "GSE11708"
"39" "GSE11758"
"40" "GSE11762"
"41" "GSE11807"
"42" "GSE11852"
"43" "GSE12029"
"44" "GSE12137"
"45" "GSE12316"
"46" "GSE12401"
"47" "GSE12402"
"48" "GSE12403"
"49" "GSE12522"
"50" "GSE12551"
"51" "GSE12619"
"52" "GSE12676"
"53" "GSE12691"
"54" "GSE12715"

"55" "GSE12729"
"56" "GSE12856"
"57" "GSE12887"
"58" "GSE12911"
"59" "GSE13383"
"60" "GSE13469"
"61" "GSE13596"
"62" "GSE13739"
"63" "GSE13783"
"64" "GSE13803"
"65" "GSE13833"
"66" "GSE13881"
"67" "GSE13913"
"68" "GSE13927"
"69" "GSE13929"
"70" "GSE13930"
"71" "GSE14091"
"72" "GSE14229"
"73" "GSE14247"
"74" "GSE14374"
"75" "GSE14420"
"76" "GSE14493"
"77" "GSE14496"
"78" "GSE8927"
"79" "GSE14646"
"80" "GSE14765"
"81" "GSE14788"
"82" "GSE1491"
"83" "GSE14961"
"84" "GSE15105"
"85" "GSE15160"
"86" "GSE15165"
"87" "GSE15189"
"88" "GSE15236"
"89" "GSE15515"
"90" "GSE15555"
"91" "GSE15577"
"92" "GSE15601"
"93" "GSE15617"
"94" "GSE15649"
"95" "GSE15680"
"96" "GSE15689"
"97" "GSE15939"
"98" "GSE16061"
"99" "GSE16143"
"100" "GSE16222"
"101" "GSE16333"
"102" "GSE16468"
"103" "GSE16469"
"104" "GSE16471"
"105" "GSE16472"
"106" "GSE16497"
"107" "GSE16557"
"108" "GSE16913"

"109" "GSE16964"
"110" "GSE17099"
"111" "GSE17159"
"112" "GSE17193"
"113" "GSE17343"
"114" "GSE17382"
"115" "GSE17464"
"116" "GSE17496"
"117" "GSE17610"
"118" "GSE17675"
"119" "GSE17824"
"120" "GSE17845"
"121" "GSE17875"
"122" "GSE18112"
"123" "GSE18217"
"124" "GSE18225"
"125" "GSE18273"
"126" "GSE18417"
"127" "GSE18624"
"128" "GSE18631"
"129" "GSE18666"
"130" "GSE18818"
"131" "GSE18960"
"132" "GSE18975"
"133" "GSE18977"
"134" "GSE18978"
"135" "GSE18979"
"136" "GSE18980"
"137" "GSE18981"
"138" "GSE18982"
"139" "GSE18983"
"140" "GSE18984"
"141" "GSE18985"
"142" "GSE18986"
"143" "GSE18987"
"144" "GSE18988"
"145" "GSE19109"
"146" "GSE19241"
"147" "GSE19252"
"148" "GSE19254"
"149" "GSE19255"
"150" "GSE19256"
"151" "GSE19258"
"152" "GSE19259"
"153" "GSE19260"
"154" "GSE19261"
"155" "GSE19262"
"156" "GSE19263"
"157" "GSE19264"
"158" "GSE19265"
"159" "GSE19266"
"160" "GSE19267"
"161" "GSE19268"
"162" "GSE19269"

"163" "GSE19271"
"164" "GSE19273"
"165" "GSE19341"
"166" "GSE19530"
"167" "GSE19603"
"168" "GSE19661"
"169" "GSE20009"
"170" "GSE20039"
"171" "GSE20221"
"172" "GSE20222"
"173" "GSE20223"
"174" "GSE20224"
"175" "GSE20226"
"176" "GSE20228"
"177" "GSE20231"
"178" "GSE20233"
"179" "GSE20256"
"180" "GSE20258"
"181" "GSE20454"
"182" "GSE20493"
"183" "GSE20556"
"184" "GSE21504"
"185" "GSE2169"
"186" "GSE21762"
"187" "GSE21765"
"188" "GSE21786"
"189" "GSE21981"
"190" "GSE22114"
"191" "GSE2218"
"192" "GSE2268"
"193" "GSE2406"
"194" "GSE2473"
"195" "GSE2538"
"196" "GSE2847"
"197" "GSE2848"
"198" "GSE3056"
"199" "GSE3326"
"200" "GSE3350"
"201" "GSE3416"
"202" "GSE3423"
"203" "GSE3424"
"204" "GSE3865"
"205" "GSE3959"
"206" "GSE4113"
"207" "GSE431"
"208" "GSE4662"
"209" "GSE4733"
"210" "GSE4847"
"211" "GSE5465"
"212" "GSE5513"
"213" "GSE5521"
"214" "GSE5522"
"215" "GSE5523"
"216" "GSE5524"

"217" "GSE5525"
"218" "GSE5526"
"219" "GSE5528"
"220" "GSE5529"
"221" "GSE5530"
"222" "GSE5533"
"223" "GSE5534"
"224" "GSE5535"
"225" "GSE5536"
"226" "GSE5537"
"227" "GSE5539"
"228" "GSE5611"
"229" "GSE5612"
"230" "GSE5614"
"231" "GSE5615"
"232" "GSE5616"
"233" "GSE5617"
"234" "GSE5618"
"235" "GSE5619"
"236" "GSE5620"
"237" "GSE5621"
"238" "GSE5622"
"239" "GSE5623"
"240" "GSE5624"
"241" "GSE5625"
"242" "GSE5626"
"243" "GSE5627"
"244" "GSE5628"
"245" "GSE5629"
"246" "GSE5630"
"247" "GSE5631"
"248" "GSE5632"
"249" "GSE5633"
"250" "GSE5634"
"251" "GSE5636"
"252" "GSE5637"
"253" "GSE5638"
"254" "GSE5639"
"255" "GSE5640"
"256" "GSE5641"
"257" "GSE5684"
"258" "GSE5685"
"259" "GSE5686"
"260" "GSE5687"
"261" "GSE5688"
"262" "GSE5696"
"263" "GSE5697"
"264" "GSE5698"
"265" "GSE5699"
"266" "GSE5700"
"267" "GSE5701"
"268" "GSE5710"
"269" "GSE5711"
"270" "GSE5712"

"271" "GSE5722"
"272" "GSE5723"
"273" "GSE5724"
"274" "GSE5725"
"275" "GSE5726"
"276" "GSE5727"
"277" "GSE5728"
"278" "GSE5729"
"279" "GSE5730"
"280" "GSE5731"
"281" "GSE5733"
"282" "GSE5734"
"283" "GSE5735"
"284" "GSE5736"
"285" "GSE5737"
"286" "GSE5738"
"287" "GSE5739"
"288" "GSE5740"
"289" "GSE5742"
"290" "GSE5744"
"291" "GSE5745"
"292" "GSE5750"
"293" "GSE5751"
"294" "GSE5759"
"295" "GSE5770"
"296" "GSE5806"
"297" "GSE6024"
"298" "GSE6025"
"299" "GSE6147"
"300" "GSE6149"
"301" "GSE6151"
"302" "GSE6153"
"303" "GSE6154"
"304" "GSE6155"
"305" "GSE6158"
"306" "GSE6160"
"307" "GSE6161"
"308" "GSE6162"
"309" "GSE6165"
"310" "GSE6166"
"311" "GSE6167"
"312" "GSE6168"
"313" "GSE6169"
"314" "GSE6171"
"315" "GSE6174"
"316" "GSE6175"
"317" "GSE6176"
"318" "GSE6177"
"319" "GSE6178"
"320" "GSE6179"
"321" "GSE6181"
"322" "GSE6203"
"323" "GSE627"
"324" "GSE629"

"325" "GSE630"
"326" "GSE631"
"327" "GSE6516"
"328" "GSE6556"
"329" "GSE6583"
"330" "GSE6638"
"331" "GSE6696"
"332" "GSE6788"
"333" "GSE6812"
"334" "GSE6826"
"335" "GSE6827"
"336" "GSE6828"
"337" "GSE6829"
"338" "GSE6830"
"339" "GSE6831"
"340" "GSE6832"
"341" "GSE6906"
"342" "GSE6954"
"343" "GSE7003"
"344" "GSE7112"
"345" "GSE7211"
"346" "GSE7227"
"347" "GSE7334"
"348" "GSE7353"
"349" "GSE7432"
"350" "GSE7631"
"351" "GSE7636"
"352" "GSE7639"
"353" "GSE7641"
"354" "GSE7642"
"355" "GSE7743"
"356" "GSE7796"
"357" "GSE7961"
"358" "GSE8248"
"359" "GSE8257"
"360" "GSE8279"
"361" "GSE8319"
"362" "GSE8365"
"363" "GSE8739"
"364" "GSE8741"
"365" "GSE8745"
"366" "GSE8785"
"367" "GSE8787"
"368" "GSE8912"
"369" "GSE8913"
"370" "GSE8925"
"371" "GSE8926"
"372" "GSE8951"
"373" "GSE8975"
"374" "GSE8994"
"375" "GSE911"
"376" "GSE9148"
"377" "GSE9201"
"378" "GSE9311"

"379" "GSE9402"
"380" "GSE9408"
"381" "GSE9597"
"382" "GSE9605"
"383" "GSE9674"
"384" "GSE9702"
"385" "GSE9719"
"386" "GSE9728"
"387" "GSE9816"
"388" "GSE9955"
"389" "GSE9956"
"390" "GSE9957"
"391" "GSE9996"
